# Establishment and validation of a nomogram with intratumoral heterogeneity derived from ^18^F-FDG PET/CT for predicting individual conditional risk of 5-year recurrence before initial treatment of nasopharyngeal carcinoma

**DOI:** 10.1186/s12885-020-6520-5

**Published:** 2020-01-15

**Authors:** Bingxin Gu, Jianping Zhang, Guang Ma, Shaoli Song, Liqun Shi, Yingjian Zhang, Zhongyi Yang

**Affiliations:** 1 Department of Nuclear Medicine, Fudan University Shanghai Cancer Center, No. 270, Dong’an Road, Shanghai, 200032 Xuhui District China; 2 Department of Oncology, Shanghai Medical College, Fudan University, Shanghai, 200032 China; 3 Center for Biomedical Imaging, Fudan University, Shanghai, 200032 China; 4 Shanghai Engineering Research Center of Molecular Imaging Probes, Shanghai, 200032 China; 5 Key Laboratory of Nuclear Physics and Ion-beam Application (MOE), Fudan University, Shanghai, 200433 China; 6 Department of Nuclear Science and Technology, Fudan University, Shanghai, 200433 China

**Keywords:** Positron emission tomography computed tomography, Nasopharyngeal carcinoma, Nomogram, Progression-free survival, Radiotherapy

## Abstract

**Background:**

Intratumoral heterogeneity has an enormous effect on patient treatment and outcome. The purpose of the current study was to establish and validate a nomogram with intratumoral heterogeneity derived from ^18^F-fluorodeoxyglucose (FDG) positron emission tomography/computed tomography (PET/CT) for prognosis of 5-Year progression-free survival (PFS) of patients with nasopharyngeal carcinoma (NPC).

**Methods:**

A total of 171 NPC patients who underwent pretreatment ^18^F-FDG PET/CT were retrospectively enrolled. Data was randomly divided into training cohort (*n* = 101) and validation cohort (*n* = 70). The clinicopathologic parameters and the following PET parameters were analyzed: maximum and mean standardized uptake value (SUVmax, SUVmean), metabolic tumor volume (MTV), total lesion glycolysis (TLG), and heterogeneity index (HI, SUVmax/SUVmean) for primary tumor and maximal neck lymph node. Cox analyses were performed on PFS in the training cohort. A prognostic nomogram based on this model was developed and validated.

**Results:**

For the primary tumor, MTV-2.5, TLG-2.5, MTV-70%, and TLG-70% were significantly correlated with PFS. For the maximal neck lymph node, short diameter and HI were significantly correlated with PFS. Among the clinicopathologic parameters, M stage was a significant prognostic factor for recurrence. In multivariate analysis, M stage (*P* = 0.006), TLG-T-70% (*P* = 0.002), and HI-N (*P* = 0.018) were independent predictors. Based on this prognostic model, a nomogram was generated. The C-index of this model was 0.74 (95% CI: 0.63–0.85). For the cross validation, the C-index for the model was 0.73 (95% CI: 0.62–0.83) with the validation cohort. Patients with a risk score of ≥111 had poorer survival outcomes than those with a risk score of 0–76 and 77–110.

**Conclusions:**

Intratumoral heterogeneity derived from ^18^F-FDG PET/CT could predict long-term outcome in patients with primary NPC. A combination of PET parameters and the TNM stage enables better stratification of patients into subgroups with different PFS rates.

## Background

Nasopharyngeal carcinoma (NPC) is a unique epithelial carcinoma, which has distinctive geographic distribution, ethnic variation, and histopathological characteristics [1]. There are approximately 129,000 newly diagnosed nasopharyngeal carcinoma and 73,000 patients die of this neoplasm worldwide in 2018 [2]. According to the WHO criteria, keratinizing squamous cell carcinoma is defined as type I, whereas types II and III refer to non-keratinizing differentiated and undifferentiated carcinomas, respectively. In regions where nasopharyngeal carcinoma is endemic, e.g. South-Eastern Asia and North Africa, non-keratinizing carcinomas comprise almost 95% of cases, which are invariably associated with Epstein-Barr virus (EBV) infection [3]. Due to the anatomic location and high radiosensitivity, radiotherapy (RT) is the primary and only curative treatment for nasopharyngeal carcinoma. Meanwhile, chemotherapy and targeted therapy also serve as pivotal advancement in the treatment of the locally advanced nasopharyngeal carcinoma [4–6]. However, the long-term prognosis remains relatively poor as the current therapeutic regimens are mainly depended on TNM stage [7–9].

Currently, the American Joint Committee on Cancer (AJCC)/Union for International Cancer Control (UICC) TNM classification is the most widely used disease staging system. As it only considers the anatomical information of tumor and ignoring the biological heterogeneity, the TNM staging system couldn’t serve as a perfect prognostic tool for estimating the risk of recurrence [10, 11]. Instead, many other factors, such as age [12], sex [13], body mass index (BMI) [14], serum lactate dehydrogenase (LDH) [10], inflammatory biomarkers [15], and pretreatment EBV DNA load [16], have been reported as individual prognostic biomarkers for survival prediction. However, these prognostic models still lack accuracy, and couldn’t directly reflect the intratumoral information, which may play a more important role in survival prediction.

Research over decades has demonstrated that intratumoral heterogeneity has an enormous effect on patient treatment and outcome [17]. ^18^F-fluorodeoxyglucose (FDG) positron emission tomography/computed tomography (PET/CT), which combined anatomical information and metabolic information, has shown value in the assessment of intratumoral heterogeneity [18–20]. Some semiquantitative parameters acquired from PET/CT, including the standardized uptake value (SUV), metabolic tumor volume (MTV), total lesion glycolysis (TLG), and heterogeneity index (HI), are demonstrated to be valuable for risk stratification and evaluation of prognosis [21–23]. Chung et al. [24] investigated the ability of MTV to predict short-term outcome in patients with NPC and found that MTV of > 40 mL was significantly correlation with poor prognosis. Chen et al. [22] revealed that TLG reduction ratio of > 0.6 during the treatment of NPC patients predicted better survival outcome. However, the value of intratumoral heterogeneity for predicting the survival outcomes in NPC patients has not been well investigated.

Given this background, we aimed (1) to determine the prognostic significance of intratumoral heterogeneity derived from PET/CT, and (2) to build a prognostic nomogram model for assessing the long-term survival outcome in patients with primary NPC.

## Methods

### Patient selection

All patients included in this analysis were diagnosed with nasopharyngeal carcinoma, and were primary treated at our institution between May 2009 and March 2014. Patients were included if they met the following criteria: 1) age > 16 and < 80 years; 2) pathology confirmed nasopharyngeal carcinoma; 3) PET/CT scans performed 4 weeks prior to treatment; and 4) stage III and IV according to the 8th edition American Joint Committee on Cancer (AJCC) guidelines [25]. We excluded patients who did not receive radiotherapy, patients with a history of other malignancies, and patients with a follow-up less than 5 years. This study was approved by the Ethical Committee at Fudan University Shanghai Cancer Center (FUSCC), and informed consent was obtained from all enrolled patients.

In our institution database of primary nasopharyngeal carcinoma patients, 171 patients were eligible for this study. Data included demographics, tumor characteristics, and treatment outcomes were retrospectively collected from the medical records. All patients were staged according to the 8th edition AJCC guidelines. Patients with metastatic disease at the time of initial diagnosis (M1 stage) were also included. Epstein-Barr virus (EBV) status was determined by testing plasma anti-EBV IgA antibodies using ELISA. EBV status was available for 62% of the patients.

### Treatment and follow-up

All patients received Intensity-Modulated Radiation Therapy (IMRT) for a cumulative dose of 66 Gy (2.2 Gy/fraction/day) in 30 fractions for T1 and T2 disease or 70.4 Gy (2.2 Gy/fraction/day) in 32 fractions for T3 and T4 lesion. According to the tumor stage and other clinical characteristics, concomitant chemotherapy or targeted therapy was also performed. Induction chemotherapy was consisted of docetaxel 75 mg/m^2^ on day 1, cisplatin 75 mg/m^2^ on day 1, and 5-Fu 500 mg/m^2^/d continuously on day 1–5. With respect to concurrent chemoradiotherapy (CCRT), cisplatin 40 mg/m^2^ was used weekly during radiation. As for adjuvant chemotherapy, cisplatin 40 mg/m^2^ on day 1–3, and docetaxel 75 mg/m^2^ on day 4 after radiation. Cetuximab was used as a targeted drug with an initial dose of 400 mg/m^2^ followed by 250 mg/m^2^ weekly for the duration of radiotherapy. Individual treatment protocol was approved by the Nasopharyngeal Carcinoma multidisciplinary team in our institution after the consultation.

After completion of radiotherapy, physical examination, imaging examination, and nasopharyngoscopy were performed every 3 months in the first 2 years, then every 6 months in the third to fifth year and once a year thereafter. Local recurrence and distant metastasis were proven by pathologic evidence or radiologic evidence. We identified treatment response according to RECIST 1.1. The following endpoints were evaluated: progression-free survival (PFS) and loco-regional control (LRC). PFS was calculated from the first day of RT to the date of disease progression or was censored at the last follow-up date. LRC was measured from the first day of RT to the date of first recurrence in the primary tumor and/or lymph node.

### PET/CT scanning procedure

^18^F-FDG PET/CT scans were performed using a Siemens biograph 16HR PET/CT scanner (Knoxville, Tennessee, USA). Patients were requested to fast at least 6 h and maintain the venous blood glucose levels under 10 mmol/L before ^18^F-FDG injection. With Explora FDG_4_ module, ^18^F-FDG was produced automatically using a Siemens CTI RDS Eclips ST cyclotron (Knoxville, Tennessee, USA) and had a radiochemical purity greater than 95%. Each patient got an injection with 7.4 MBq/kg ^18^F-FDG. After approximately 63.17 ± 7.15 (50–77) mins, PET/CT images were acquired. Helical CT was performed before PET with a scanning range from the proximal thighs to head. The CT acquisition parameters were as follows: tube voltages:120 kV, tube current: 80 ~ 250 mA, slice thickness: 5.0 mm, pitch: 1.0 mm, rotation time: 0.5 s. PET were acquired with 2 ~ 3 min per table position. PET image data sets were reconstructed iteratively using an ordered-subset expectation maximization iterative reconstruction (OSEM) by applying CT data for attenuation correction. The reconstruction parameters were as follows: iterations: 4, subsets: 8, pixel size: 4.0 × 4.0 mm, zoom: 1.0, FWHM: 6.0 mm, and slice thickness: 5.0 mm. Fusion images were reviewed and manipulated on a multimodality computer platform (Syngo, Siemens, Knoxville, Tennessee, USA). Two experienced radiologists analyzed and interpreted the images independently, and the reviewers reached a consensus in case of inconsistency.

For quantitative analysis, maximum and mean of standardized uptake value (SUV) normalized to body weight were manually computed for primary tumor (SUVmax-T, SUVmean-T) and neck lymph node with maximum volume (SUVmax-N, SUVmean-N) by drawing a region of interest (ROI). Meanwhile, metabolic tumor volume (MTV) was recorded at the absolute SUV threshold of 2.5 and the relative SUVmax threshold of 70%. Total lesion glucose (TLG) was calculated according to the formula: TLG = SUVmean × MTV. To evaluate intratumoral heterogeneity, heterogeneity index (HI) [19] was obtained by dividing SUVmax by SUVmean (absolute SUV threshold of 2.5) for primary lesion and nodal disease.

### Statistical analysis

The entire cohort was divided into a training cohort (*n* = 101) and a validation cohort (*n* = 70). The following parameters were assessed to identify predictors of recurrence: age, gender, EBV statue, histology, tumor staging, treatment, and PET parameters. Frequencies with percentages were used to describe categorical variables while medians with ranges were used for continuous characteristics. The differences of these parameters between these two cohorts were calculated. Mann-Whitney tests were used to compare the continuous variables, and Fisher’s exact tests were used to compare the categorical data. The survival analyses were performed using the Kaplan-Meier method, and a two-sided log-rank test was used to compare groups.

The predictive model was constructed as suggested in the TRIPPOD statement [26]. To develop a robust and well-calibrated nomogram predicting the risk of recurrence, a cox regression model was built using a training cohort of 101 patients and validated with a cohort of 70 patients. Firstly, a univariate Cox analysis was performed to assess relationships between risk factors and recurrence using the training cohort. The Harrell’s C-index was computed for the factor with significance of *P* < 0.05. Then, predictors were determined using the factors with significance of *P* < 0.1 and with highest C-index after univariate analyses, and the multivariate Cox regression model was developed with backward elimination. The Harrell’s C-index, the constant, and the standardized coefficient of the prognostic model were calculated. Lastly, based on this model, a nomogram was built to predict the individual conditional risk of 5-year recurrence.

To estimate the accuracy of the model, internal validation was performed by bootstrap algorithm, in which 1000 replications were constructed randomly, and the adjusted C-index and corresponding 95% confidence intervals were also computed. The calibration plot comparing the nomogram predicted versus observed probability was used to assess the accuracy. To test for generalizability, the developed nomogram derived from the training cohort was tested with the validation cohort. Three prognostic groups were created by categorizing the prognostic index computed from the model at the 55th and 89th percentiles using the X-tile software [27]. These groups were called low-, intermediate- and high-risk groups. The same cut-off was applied in the validation cohort. All statistical tests were two-sided, and the *p* < 0.05 was considered statistically significant. All analyses were performed using SPSS (version 22.0; IBM Inc., New York, USA) and R version 3.5.3 (http://cran.r-project.org/mirrors.html).

## Results

### Patient characteristics

The characteristics of training and validation cohort are presented in Table 1. The median age at diagnosis was 43 (16–78) years in the training cohort and 46 (17–70) years in the validation cohort. The majority were male (80.20 and 71.43%, respectively) and typically had non-keratinizing undifferentiated NPC (69.31 and 78.57%, respectively) in these two cohorts. EBV status was available for 62% of the patients. Pretreatment EBV antibody was positive in 45.54% (46/101) and 55.71% (39/70) patients in the training and validation cohort, respectively. Given that no significant difference in PFS between negative and unknown EBV status was identified, these two statuses were combined into one group. The M1 stage were available for 6.93% (7 of 101) and 5.71% (4 of 70) of patients in the training and validation cohort, respectively. The 5-year PFS rate for training and validation cohort were 79.21% (80 of 101) and 68.57% (48 of 70 patients), respectively.

**Table 1:** Patient characteristics in the training and validation cohorts

Characteristics	Training cohort (*N* = 101)	Validation cohort (*N* = 70)	*P* value^a^
Age (years), median (min-max)	43 (16–78)	46 (17–70)	0.61
Gender, n (%)			0.18
Male	81 (80.20)	50 (71.43)	
Female	20 (19.80)	20 (28.57)	
EBV antibody, n (%)			0.19
Positive	46 (45.54)	39 (55.71)	
Negative/Unknown	55 (54.46)	31 (44.29)	
Histology, WHO Type^b^, n (%)			0.24
I/II	31 (30.69)	15 (21.43)	
III	70 (69.31)	55 (78.57)	
BMI (Kg/m^2^), mean (min-max)	23.14 (17.30–32.91)	22.30 (15.40–29.05)	0.09
T stage, n (%)			0.88
T1	31 (30.69)	21 (30.00)	
T2	13 (12.87)	8 (11.43)	
T3	45 (44.56)	33 (47.14)	
T4	12 (11.88)	8 (11.43)	
N stage, n (%)			0.81
N0	5 (4.95)	3 (4.29)	
N1	21 (20.79)	13 (18.57)	
N2	57 (56.44)	42 (60.00)	
N3	18 (17.82)	12 (17.14)	
M stage, n (%)			0.75
M0	94 (93.07)	66 (94.29)	
M1	7 (6.93)	4 (5.71)	
TNM stage (AJCC), n (%)			0.48
III	67 (66.34)	50 (71.43)	
IV a	27 (26.73)	16 (22.86)	
IV b	7 (6.93)	4 (5.71)	
Concomitant systemic treatment with IMRT, n (%)			0.92
None	6 (5.94)	1 (1.43)	
Chemotherapy	80 (79.21)	61 (87.14)	
Targeted Therapy	15 (14.85)	8 (11.43)	
Treatment results			
Median Follow-up, months, (min-max)	63 (4–109)	67.5 (3–113)	0.57
5-year LRC rate	89.11%	87.14%	0.29
5-year PFS rate	79.21%	68.57%	0.12

^a^Statistical comparisons between the training and validation cohorts were computed using the Mann–Whitney U test for continuous variables and χ^2^ test for categorical variables. A *P*-value of < 0.05 indicates a significant difference

^b^WHO Type I = keratinizing, WHO Type II = non-keratinizing (differentiated), WHO Type III = non-keratinizing (undifferentiated)

Abbreviations: *WHO* World Health Organization, *AJCC* American Joint Committee on Cancer, *IMRT* Intensity-modulated radiation therapy, *LRC* Loco-regional control, *PFS* Progression-free survival

### Identification of the cox models and nomograms to predict PFS in the training cohort

**Fig. 1:**
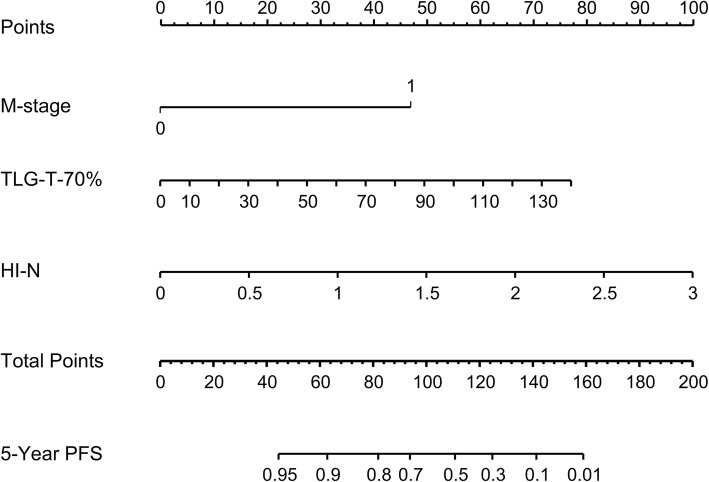
Nomogram-1 for 5-Year PFS based on the training cohort. This nomogram was based on M-stage, TLG-T-70%, and HI-N. For each patient, the total score was the sum of points of these three factors, which were respectively identified on the points scale. The 5-Year PFS probability of each patient was then determined on the total points scale. Abbreviations: PFS, progression-free survival; TLG-T, total lesion glucose of tumor; HI-N, heterogeneity index of maximal neck lymph node

For calculating SUVmean, MTV and TLG, we compared the SUV threshold of absolute value 2.5 and relative value of 40, 50, 60, and 70%. However, among these relative thresholds, only 70% showed better correlation with PFS in univariate analysis with lower *p* values. Therefore, we only displayed the SUV threshold with absolute value of 2.5 and relative value of 70% in this study. Table 2 shows the results of Cox regression analyses used to identify predictors of PFS. Univariate analysis showed that tumor stage and six PET parameters were significantly associated with PFS. For the primary tumor, MTV and TLG with absolute threshold of 2.5 and relative threshold of 70% were significantly correlated with PFS, with a C-index of 0.61, 0.46, 0.62 and 0.60, respectively. For the maximal neck lymph node, short diameter and HI were significantly correlated with PFS, with a C-index of 0.63 and 0.66, respectively. Notably, SUVmax and SUVmean for both the primary tumor and maximal neck lymph node showed no significantly association with PFS (Additional file 1: Table S1). HI of the primary tumor was also not significantly correlated with PFS. As showed in Table 3, multivariate analysis yielded three statistically significant predictors: M stage (HR, 6.44; 95% CI, 1.72–24.08; *p* = 0.006), TLG of the tumor with a relative threshold of 70% (HR, 1.02; 95% CI, 1.01–1.04; *p* = 0.002), and HI of the maximal neck lymph node (HR, 3.23; 95% CI, 1.22–8.54; *p* = 0.018). The C-index of this model (model 1) was 0.74 (95% CI: 0.63–0.85). The point value assigned to each factor was proportional to the hazard ratio derived from its own β-coefficients determined by the Cox regression analysis. Nomogram-1 was constructed based on this Cox regression model (Fig. 1). Nomogram-2 (Additional file 1: Fig. S1) was constructed with M stage and TLG of the tumor with a relative threshold of 70%, yielding a model (model 2) with C-index of 0.64 (95% CI: 0.50–0.78).

**Table 2:** Univariate analysis for PFS in the training cohort

Parameters	HR [95% CI]	C-index	*P* value
Clinical parameters			
Age (years)	–	–	0.74
Gender			
Male	–	–	0.81
Female			
EBV antibody			
Positive	–	–	0.49
Negative/Unknown			
Histology, WHO Type			
I/II	–	–	0.86
III			
BMI (Kg/m^2^), mean (min-max)	–	–	0.54
T stage			
T1	–	–	0.72
T2			
T3			
T4			
N stage			
N0	–	–	0.91
N1			
N2			
N3			
M stage			
M0	1	0.56	0.008
M1	5.247 [1.532–17.973]		
Stage (AJCC)			
III	1	0.61	0.02
IV a	1.615 [0.626–4.165]		0.32
IV b	6.161 [1.704–22.278]		0.006
Concomitant systemic treatment with IMRT			
None	–	–	0.16
Chemotherapy			
Targeted Therapy			
PET Parameters			
MTV-T-2.5	1.025 [1.007–1.043]	0.61	0.006
TLG-T-2.5	1.004 [1.001–1.007]	0.46	0.009
MTV-T-70%	1.118 [1.012–1.236]	0.62	0.029
TLG-T-70%	1.016 [1.004–1.028]	0.60	0.009
Diameter-N	1.565 [1.005–2.436]	0.63	0.047
HI-N	2.858 [1.274–6.407]	0.66	0.011

For PET parameters, data are only provided for absolute and relative thresholds with the highest C-index and *P* < 0.05. A *P*-value of < 0.05 indicates a significant difference

Abbreviations: *PFS* Progression-free survival, *HR* Hazard ratio, *CI* Confidence interval, *WHO* World Health Organization, *AJCC* American Joint Committee on Cancer, *IMRT* Intensity-modulated radiation therapy, PET Positron emission tomography, *MTV-T* Metabolic tumor volume of tumor, *TLG-T* Total lesion glucose of tumor, *HI-N* Heterogeneity index of maximal neck lymph node

**Table 3:** Significant predictors of PFS in multivariate analysis in the training cohort

Parameters	Multivariate Cox analysis					Cox model bootstrap validation (1000 samples)		
	HR [95% CI]	*P*	C-index	Standardized regression coefficient	SE	HR [95% CI]	*P*	C-index
M stage	6.44 [1.72–24.08]	0.006	0.74	1.86	0.67	6.35 [1.72–23.43]	0.006	0.72
TLG-T-70%	1.02 [1.01–1.04]	0.002		0.02	0.01	1.02 [1.01–1.04]	0.004	
HI-N	3.23 [1.22–8.54]	0.018		1.17	0.50	3.70 [1.59–8.61]	0.025	

Abbreviations: *PFS* Progression-free survival, *HR* Hazard ratio, *CI* Confidence interval, *SE* Standard error, *TLG-T* Total lesion glucose of tumor, *HI-N* Heterogeneity index of maximal neck lymph node

### Internal validations of the prognostic model (model 1)

**Fig. 2:**
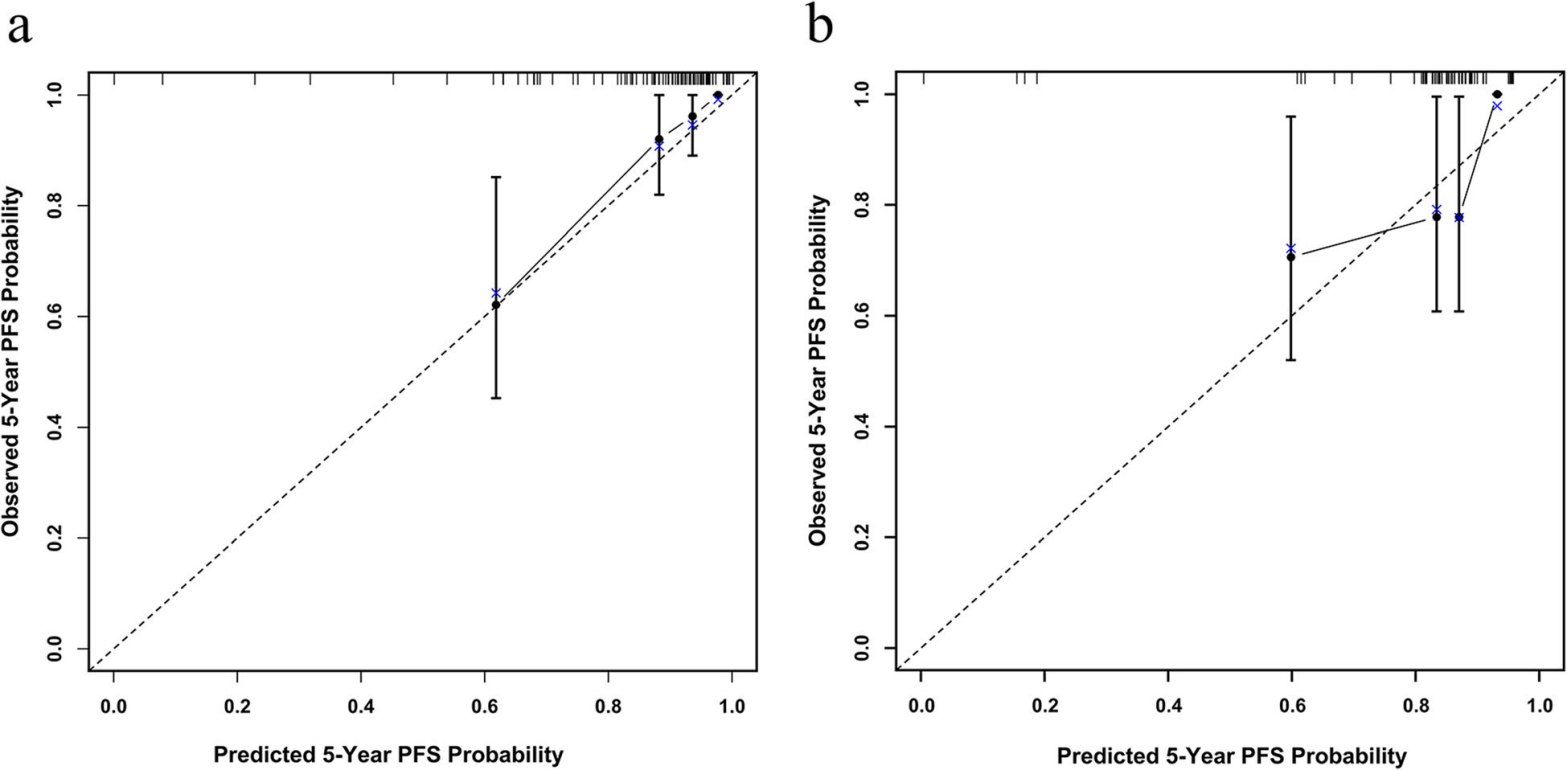
Calibration plots for predicting 5-Year PFS of patients with NPC in training cohort (**a**) and validation cohort (**b**). Nomogram-1 predicted 5-Year PFS is plotted on the x-axis, and observed 5-Year PFS is plotted on the y-axis. Dashed lines represent the perfect calibration models, in which the predicted probabilities are identical to the observed probabilities. Abbreviations: PFS, progression-free survival; NPC, nasopharyngeal carcinoma

**Fig. 3:**
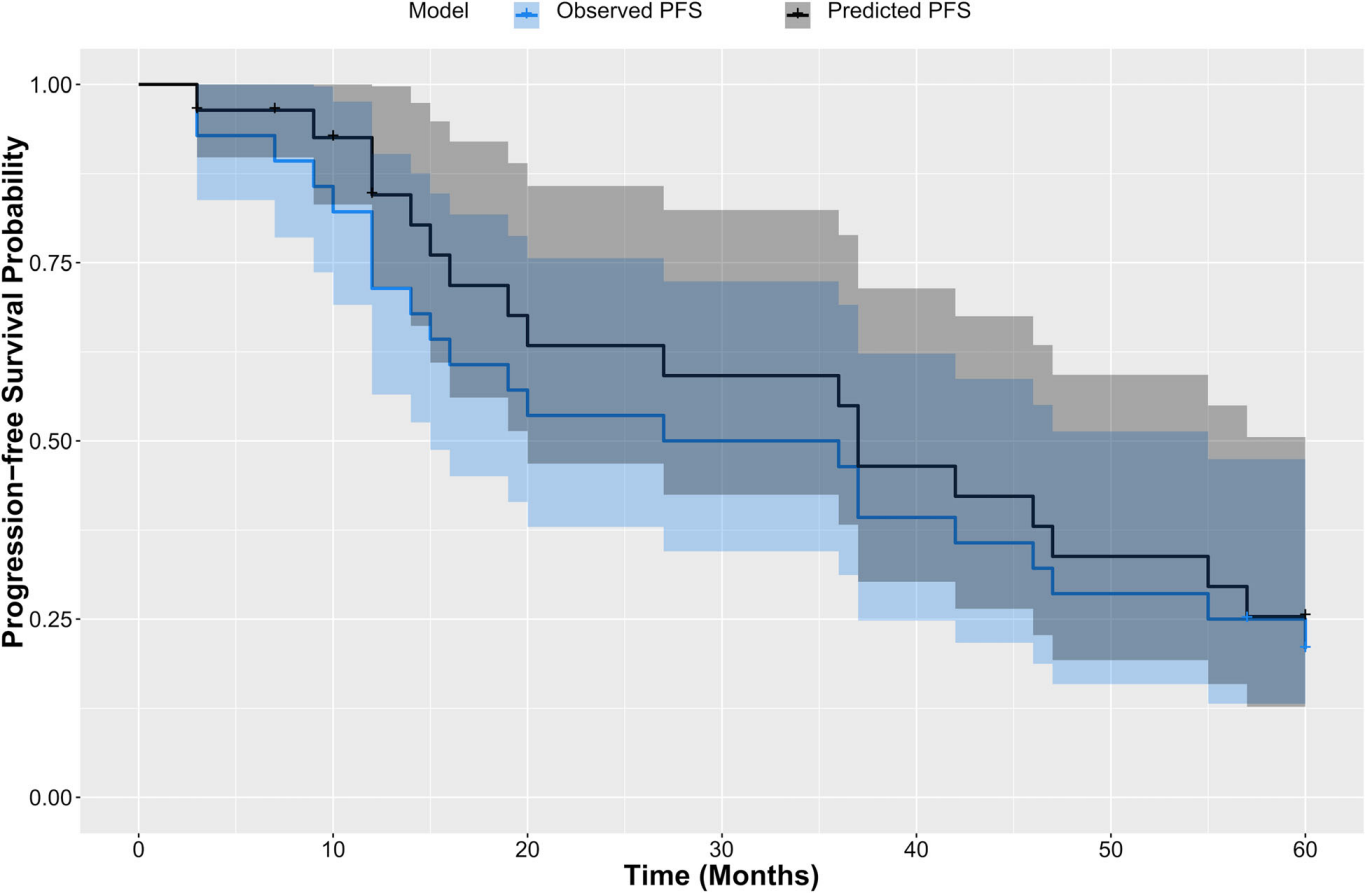
Predicted and observed Kaplan–Meier curves of PFS for the validation cohort based on model 1. The blue line indicates the observed PFS for the validation cohort, and the blue area corresponds to the 95% CI. The black line indicates the predicted PFS when applying the prognostic model to the patients of the validation cohort, and the grey area corresponds to the 95% CI. Abbreviations: PFS, progression-free survival; CI, confidence interval

Bootstrap resampling and cross validation were performed for internal validation. After bootstrap resampling with 1000 repetitions, the corrected C-index for the model was 0.72 (95% CI: 0.61–0.83). For the cross validation, the C-index for the model was 0.73 (95% CI: 0.62–0.83) with the validation cohort. Figure 2 shows the calibration plots of the nomogram for the training and validation cohorts. They all exhibited superb agreement between the prediction according to the nomogram and actual observation. The comparison between the predicted and observed Kaplan-Meier curves of PFS for the validation cohort is presented in Fig. 3.

### Identification risk of individual patients

**Fig. 4:**
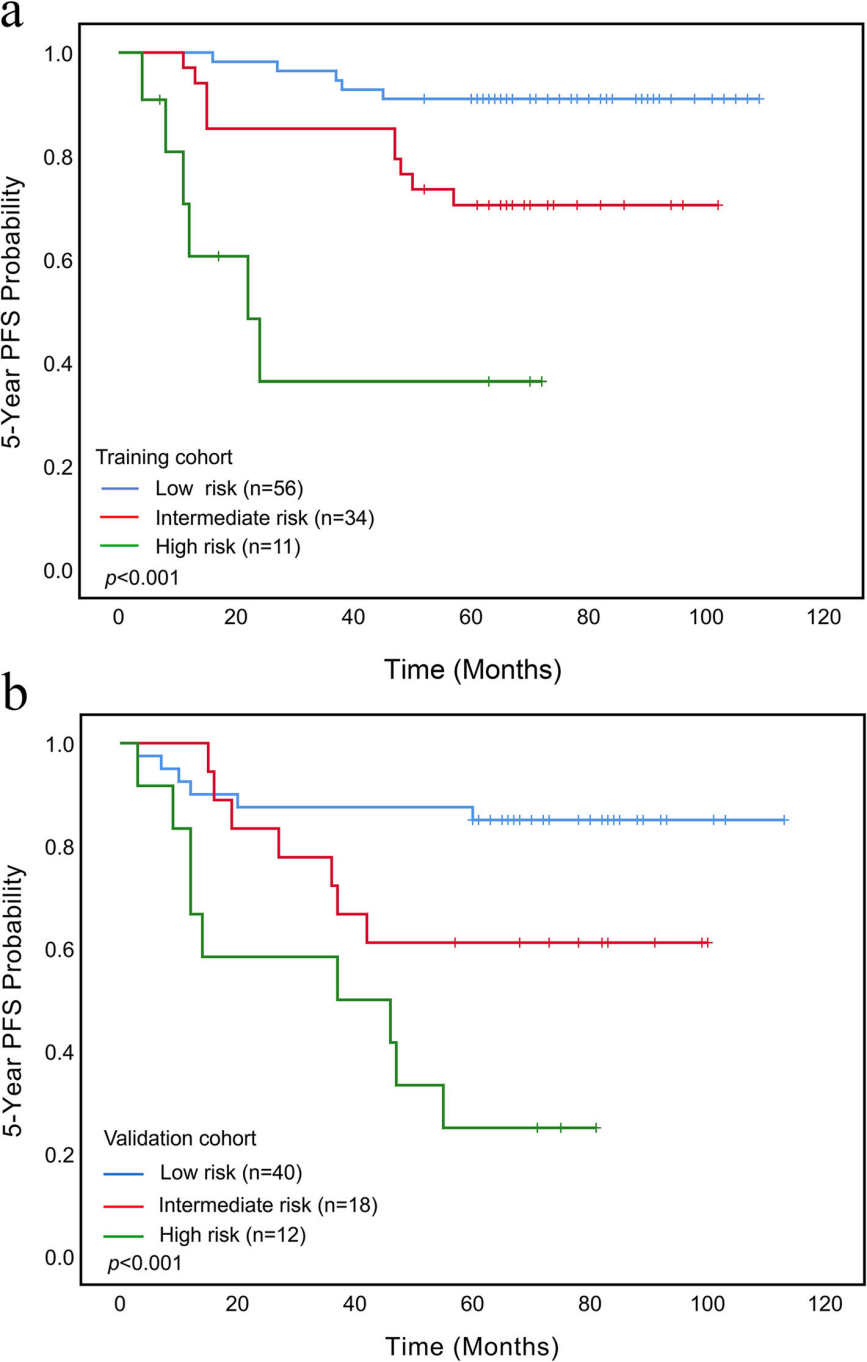
Kaplan-Meier curves of risk group stratification for 5-Year progression-free survival (PFS). Nomogram-1 risk group stratification for the 55 and 89 percentiles are shown for the training cohort (**a**) and the validation cohort (**b**)

The prognostic index was computed for each patient. Based on the cut-off computed from the training cohort, three prognostic groups (high risk, intermediate risk, and low risk) were created for both the training cohort and the validation cohort. For model 1, the 5-Year PFS rates of the three risk subgroups with risk scores of 0–76, 77–110, and ≥ 111 were 91.07, 70.59, and 45.45% for the training cohort and 85, 61.11, and 25% for the validation cohort, respectively (Additional file 1: Table S2). For model 2, the 5-Year PFS rates of the three risk subgroups with risk scores of 0–18, 19–74, and ≥ 75 were 85.71, 76.47, and 54.55% for the training cohort and 86.11, 58.62, and 0% for the validation cohort, respectively (Additional file 1: Table S3). As showed in Fig. 4, Kaplan-Meier curves of PFS for both the training cohort and the validation cohort based on model 1 revealed significantly outcomes for these three risk groups (*p* < 0.001). As showed in Additional file 1: Fig. S2, Kaplan-Meier curves of PFS for both the training cohort and the validation cohort based on model 2 also revealed significantly outcomes for these three risk groups (*p* < 0.01 for training cohort and *p* < 0.001 for validation cohort).

## Discussion

Our research demonstrated that M stage, TLG of the tumor with a relative threshold of 70%, and HI of the maximal neck lymph node were independent prognostic factors for PFS. We established two models based on M stage, TLG of the tumor with a relative threshold of 70%, and HI of the maximal neck lymph node (model 1) and M stage, TLG of the tumor with a relative threshold of 70% (model 2), respectively. The resulting nomogram-1 based on model 1 showed excellent discriminative capability (0.74; 95% CI, 0.63–0.85), while the nomogram-2 based on model 2 showed a less powerful discriminative capability (0.64; 95% CI, 0.50–0.78). Furthermore, we employed the nomogram-1 to generate risk stratifications, and as excepted, the proposed risk groups significantly discriminated the risk of 5-Year PFS in patients with primary NPC.

Compared with previous prognostic models [28–30], a major strength of our model is that intratumoral heterogeneity was taken into account. The formulation of intratumoral heterogeneity is caused by the genetic instability, which may contribute to the drug resistance and treatment failure [17, 31, 32]. Previous researches have demonstrated that the uptake of ^18^F-FDG in tumor cells can reflect the intratumoral heterogeneity by exhibiting variations in glucose metabolism of different tumor regions, and the relevant cellular and molecular characteristics are necrosis, fibrosis, hypoxia, and expression of specific receptors [18–20]. Several heterogeneity indices derived from PET/CT have been proposed, including SUVmean divided by the SUVmax [19] and linear regression slope of MTV [18]. Some other researchers define the heterogeneity index by radiomics [33, 34] and textural analysis [35, 36] of PET/CT. However, the computing method of linear regression slope of MTV varied among different researchers, and it needs more data to process [18, 37, 38]. Xu H et al. [33] proposed a data-driven approach to identify intratumoral heterogeneity of ^18^F-FDG PET/CT imaging, and constructed multiregional radiomics biomarkers to predict the PFS of NPC patients. Chan SC et al. [35] determined intratumoral heterogeneity using histogram analysis, the normalized gray-level co-occurrence matrix, and the neighborhood gray-tone difference matrix. These two methods all performed well in risk stratification of NPC patients. However, the radiomics and textural analysis are more complex, which demand special workstation and professionals, and they are not feasible integrated into clinical practice. Thus, we choose the SUVmean divided by the SUVmax, which is easier to handle, as the intratumoral heterogeneity in this study.

Previous studies have demonstrated that the intratumoral heterogeneity derived from PET/CT can serve as a prognostic biomarker for treatment outcome in pancreatic cancer [18], uterine leiomyosarcoma [20], head and neck squamous cell carcinoma [23], esophageal cancer [36], oral cavity cancer [37], and epithelial ovarian cancer [38]. Our results showed that the HI of the maximal neck lymph node was significantly associated with the long-term PFS in patients with primary NPC (HR, 3.23; 95% CI, 1.22–8.54; *p* = 0.018). Notably, the HI of the primary tumor was found to have no significant correlation with treatment outcome in this study. This may be caused by the TNM staging system that plenty of T1 patients (30.69%) were enrolled. In a newly published research [34], Peng et al. demonstrated that the intratumoral heterogeneity of primary tumor, derived from PET/CT utilizing the deep learning method, could reliably predict the response to induction chemotherapy in patients with advanced NPC. However, the percentage of T1 stage patients in this study was only 5.1%. Therefore, the prognostic significance of intratumoral heterogeneity of primary tumor needs further investigation among the low T stage and high N stage patients with primary NPC.

Among other PET/CT parameters, TLG is regarded as a promising predictor for treatment response. The prognostic value of TLG is greater than MTV and SUV [22, 39, 40]. However, most of the researches only provided the cut-off value of TLG, and the cut-off value varied in different researches [30, 39, 40]. It is not convenient in clinical practice. Our study showed that TLG was also an independent factor for predicting treatment response in patients with primary NPC (HR, 1.02; 95% CI, 1.01–1.04; *p* = 0.002). And we offered a scoring scale for each absolute value of TLG, which was exhibited in the nomogram (Fig. 1). Some investigations suggest MTV and SUV could be value in prognosis. Although our result showed that MTV of primary tumor was significantly correlated with PFS in univariate analysis, it could not serve as an independent predictor. Furthermore, neither the SUVmax nor SUVmean of primary or the maximal neck lymph node were associated with treatment response. Nevertheless, SUV could be more valuable in post-treatment scans of NPC patients [41].

According to the 8th edition AJCC/UICC staging system [25], stage IVB is classified as M1 with any T and any N. There are approximately 5–8% of NPC patients have distant metastasis at first diagnosis. ^18^F-FDG PET/CT is superior to conventional imaging modalities for detecting distant metastasis, and it is recommended for patients of NPC with a high risk of distant metastasis at initial diagnosis [42–44]. However, few studies have investigated the prognostic value of combing the M1 stage and PET/CT parameters in patients of primary NPC. Though distance metastasis is associated with poor survival, there are still a small proportion of patients with M1 can achieve complete response. If the therapeutic regimens are solely depended on TNM stage, it may cause unnecessary treatment and finical burden. Thus, we proposed the risk stratification based on the prognostic model (model 1), which established by M stage, TLG of the tumor with a relative threshold of 70%, and HI of the maximal neck lymph node, to identify the risk of individual patients. This stratification could significantly discriminate the survival outcomes for the three risk subgroups in patients with primary NPC. Patients with a score of more than 111 had poorer survival outcomes than those with a score of 0–76 or 77–110 (*p* < 0.001).

Our study had some limitations. First, Epstein-Barr virus (EBV) status was determined by testing plasma anti-EBV IgA antibodies rather than plasma EBV DNA levels, and EBV status was missing for 38% of the patients, which might limit the accuracy of statistical analysis. This may be caused by our basic national conditions as a developing country and the new technology popularized relatively late in our center. Second, the endpoint of this study was not overall survival (OS). This is due to the 5-Year OS rate of patients with NPC is high in our center. Third, our data were only obtained from a single center. For internal validation, bootstrap resampling and cross validation were performed, and the results showed the satisfactory fitting of the established models. Nevertheless, our model needs to be validated by other medical centers.

## Conclusions

In summary, our data indicated that M stage, TLG-T-70%, and HI-N were valuable in predicting long-term PFS before initial treatment in patients with NPC. We developed a novel prognostic model combing PET/CT parameters and TNM stage for predicting 5-Year PFS, and established a nomogram (nomogram-1) to identify patients with a high risk of recurrence and accordingly optimize their therapeutic regimens.

## Supplementary information

**Additional file 1: Table S1.** Univariate analysis of PET Parameters for PFS in the training cohort. **Table S2.** Risk-group based on nomogram-1 for 5-Year progression-free survival (PFS) in the training and validation cohorts. **Table S3.** Risk-group based on nomogram-2 for 5-Year progression-free survival (PFS) in the training and validation cohorts. **Figure S1.** Nomogram-2 for 5-Year PFS based on the training cohort. **Figure S2.** Kaplan-Meier curves of risk group stratification for 5-Year progression-free survival (PFS).

## Data Availability

The datasets used and analyzed during the current study are available from the corresponding author on reasonable request.

## Abbreviations

^18^F-FDG: ^18^F-fluorodeoxyglucose
AJCC: American Joint Committee on Cancer
BMI: Body mass index
CCRT: Concurrent chemoradiotherapy
EBV: Epstein-Barr virus
HI: Heterogeneity index
IMRT: Intensity-Modulated Radiation Therapy
LDH: Lactate dehydrogenase
LRC: Loco-regional control
MTV: Metabolic tumor volume
NPC: Nasopharyngeal carcinoma
PET/CT: Positron emission tomography/computed tomography
PFS: Progression-free survival
ROI: Region of interest
RT: Radiotherapy
SUV: Standardized uptake value
TLG: Total lesion glycolysis
UICC: Union for International Cancer Control
