# Supplementary material for: Establishment and validation of a nomogram with intratumoral heterogeneity derived from 18F-FDG PET/CT for predicting individual conditional risk of 5-year recurrence before initial treatment of nasopharyngeal carcinoma
Source: BMC Cancer. 2020 Jan 15;20:37. doi: 10.1186/s12885-020-6520-5 (PMC6964088; doi:10.1186/s12885-020-6520-5)
Supplement: Supplementary file 1 — Additional file 1: Table S1. Univariate analysis of PET Parameters for PFS in the training cohort. Table S2. Risk-group based on nomogram-1 for 5-Year progression-free survival (PFS) in the training and validation cohorts. Table S3. Risk-group based on nomogram-2 for 5-Year progression-free survival (PFS) in the training and validation cohorts. Figure S1. Nomogram-2 for 5-Year PFS based on the training cohort. Figure S2. Kaplan-Meier curves of risk group stratification for 5-Year progression-free survival (PFS). [file 12885_2020_6520_MOESM1_ESM.docx]

**Table S1** Univariate analysis of PET Parameters for PFS in the training cohort

| Parameters | HR [95% CI] | C-index | *P* value |
| --- | --- | --- | --- |
| SUVmax-T | 1.027 [0.940-1.122] | 0.54 | 0.556 |
| SUVmean-T-2.5 | 1.003 [0.693-1.452] | 0.54 | 0.988 |
| MTV-T-2.5 | 1.025 [1.007-1.043] | 0.61 | 0.006 |
| TLG-T-2.5 | 1.004 [1.001-1.007] | 0.46 | 0.009 |
| SUVmean-T-70% | 1.035 [0.930-1.151] | 0.54 | 0.534 |
| MTV-T-70% | 1.118 [1.012-1.236] | 0.62 | 0.029 |
| TLG-T-70% | 1.016 [1.004-1.028] | 0.60 | 0.009 |
| HI-T | 1.380 [0.616-3.091] | 0.54 | 0.434 |
| Diameter-N | 1.565 [1.005-2.436] | 0.63 | 0.047 |
| SUVmax-N | 1.066 [0.996-1.142] | 0.64 | 0.067 |
| SUVmean-N-2.5 | 1.176 [0.981-1.410] | 0.64 | 0.079 |
| MTV-N-2.5 | 1.013 [0.984-1.044] | 0.65 | 0.384 |
| TLG-N-2.5 | 1.000 [0.996-1.005] | 0.65 | 0.829 |
| SUVmean-N-70% | 1.078 [0.993-1.172] | 0.64 | 0.074 |
| MTV-N-70% | 1.046 [0.932-1.175] | 0.66 | 0.445 |
| TLG-N-70% | 1.001 [0.989-1.013] | 0.67 | 0.891 |
| HI-N | 2.858 [1.274-6.407] | 0.66 | 0.011 |

Abbrevations: PET, positron emission tomography; PFS, progression-free survival, HR, hazard ratio; CI, confidence interval; T, tumor; N, maximal neck lymph node; SUVmax, maximum of standardized uptake value; SUVmean, mean of standardized uptake value; MTV, metabolic tumor volume; TLG, total lesion glucose; HI, heterogeneity index.

**Table S2** Risk-group based on nomogram-1 for 5-Year progression-free survival (PFS) in the training and validation cohorts

| Risk-group by nomogram |  |  | Training (n=101) | |  | Validation (n=70) | |
| --- | --- | --- | --- | --- | --- | --- | --- |
|  | Cut-off | Range | n | Rate (%) |  | n | Rate (%) |
| Low | 55% | 0-76 | 56 | 91.07 |  | 40 | 85 |
| Intermediate | 89% | 77-110 | 34 | 70.59 |  | 18 | 61.11 |
| High | — | ≥111 | 11 | 45.45 |  | 12 | 25 |

**Table S3** Risk-group based on nomogram-2 for 5-Year progression-free survival (PFS) in the training and validation cohorts

| Risk-group by nomogram |  |  | Training (n=101) | |  | Validation (n=70) | |
| --- | --- | --- | --- | --- | --- | --- | --- |
|  | Cut-off | Range | n | Rate (%) |  | n | Rate (%) |
| Low | 55% | 0-18 | 56 | 85.71 |  | 36 | 86.11 |
| Intermediate | 89% | 19-74 | 34 | 76.47 |  | 29 | 58.62 |
| High | — | ≥75 | 11 | 54.55 |  | 5 | 0 |





**Figure S1** Nomogram-2 for 5-Year PFS based on the training cohort. This nomogram was based on M-stage and TLG-T-70%. For each patient, the total score was the sum of points of these two factors, which were respectively identified on the points scale. The 5-Year PFS probability of each patient was then determined on the total points scale. Abbreviations: PFS, progression-free survival; TLG-T, total lesion glucose of tumor.


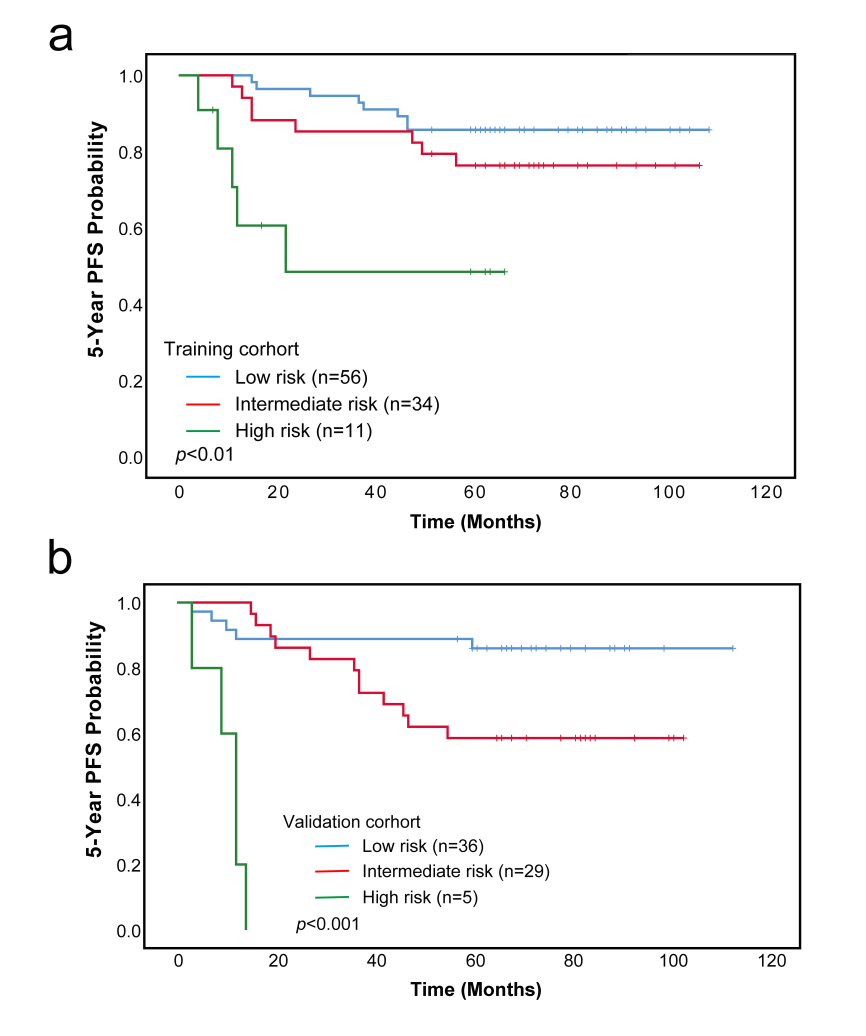


**Figure S2** Kaplan-Meier curves of risk group stratification for 5-Year progression-free survival (PFS). Nomogram-2 risk group stratification for the 55 and 89 percentiles are shown for the training cohort (a) and the validation cohort (b).
